# Diagnostic utility of nasal methicillin-resistant *Staphylococcus aureus* polymerase chain reaction testing in head and neck infection

**DOI:** 10.1017/ash.2024.376

**Published:** 2024-09-16

**Authors:** Jake Smith, Bailee Cummings, Ryan K. Dare

**Affiliations:** 1 Department of Internal Medicine, University of Arkansas for Medical Sciences, Little Rock, AR, USA; 2 Department of Internal Medicine, Tulane University, New Orleans, LA, USA; 3 Division of Infectious Diseases, University of Arkansas for Medical Sciences, Little Rock, AR, USA

## Abstract

In this retrospective study of adult inpatients who underwent an ear, nose, and throat (ENT) surgery with operative cultures and collection of nasal methicillin-resistant *Staphylococcus aureus* (MRSA) polymerase chain reaction (PCR), we found that MRSA nasal PCR demonstrated 100% sensitivity and a negative predictive value (NPV) of 100% when compared to operative cultures.

## Introduction

Infections due to methicillin-resistant *Staphylococcus aureus* (MRSA) are associated with increased hospital costs, increased hospital length of stay, and increased mortality.^1–3^ The Infectious Diseases Society of America clinical practice guidelines for severe skin and soft tissue infections (SSTIs) recommend empiric coverage with antibiotics that are active against MRSA, such as vancomycin. However, over-utilization of vancomycin can lead to unnecessary toxicities, such as acute kidney injury, and drive the emergence of antibiotic resistance, as seen with vancomycin-resistant Enterococcus.^4^

*Staphylococcus aureus* colonization of the nares can be determined by nucleic acid detection via polymerase chain reaction (PCR). Genetic information for methicillin resistance is carried on one of many cassette structures, such as MecA, which allow transmission of methicillin resistance genes from one organism to another.^5^ PCR testing can detect the presence of these cassette structures which can infer that the organism has the capability to be methicillin-resistant. In recent years, nasal swab PCR for MRSA has proven to have a remarkable negative predictive value (NPV) for MRSA pneumonia (96.5%) and thus has become a useful tool in deescalating vancomycin.^6,7^

*Staphylococcus aureus* is commonly found in the nares of colonized patients. However, with the exception of facial SSTIs, MRSA is a rare cause of head and neck infections.^8^ Current guidelines for SSTI advise that empiric coverage should be considered for MRSA in purulent infections but makes no recommendations regarding nasal MRSA PCR testing.^9^ In the present study, the sensitivity, specificity, NPV, and positive predictive value (PPV) of MRSA nasal PCR for detecting presence of MRSA from operating room (OR) cultures obtained during ear, nose, and throat (ENT) procedures were retrospectively examined.

## Methods

This is a retrospective study of the diagnostic characteristics of nasal MRSA PCR in patients with head and neck infections who underwent ENT surgery. Patient electronic medical record data included in the initial cohort were collected from a university-affiliated tertiary care center in Arkansas from January 1, 2014, through September 1, 2022. This study was reviewed and approved by the UAMS institutional review board.

Inclusion criteria were adult patients who had a single admission in which the following were collected: (a) nasal MRSA PCR testing, (b) surgery with our otolaryngologists, and (c) had OR cultures obtained during surgery. Patients <18 years old or patients with an infection from another identifiable source were excluded.

Data collected included age, gender, selected comorbid conditions, date of surgical procedure, type of surgical procedure, date of PCR collection, result of PCR test, indication for operation, date of OR culture, and results of OR culture during the hospitalization.

The index test was a nasal swab MRSA PCR, performed by the bedside nurse. PCR was performed using the GeneProof MRSA PCR kit (GeneProof, Brna, Czechia). Nasal carriage of MRSA was reported as positive or negative.

The reference standard was OR culture data. The specimen was obtained during the surgical procedure and placed in a sterile container. It was then processed by the laboratory. Cultures were considered positive for MRSA if any amount of growth was reported on the culture results.

The results of the MRSA PCR were compared to presence or absence of MRSA from the OR culture. From this, the sensitivity, specificity, PPV, and NPV were calculated. Nonparametric continuous variables were summarized as the mean values, standard deviations with confidence intervals. Categorical variables were summarized as the counts and percentages. Statistical operations were performed using Excel software (Windows office).

## Results

A total of 7,782 patients underwent surgery with our otolaryngologists during our study period. Nine hundred (11.6%) patients undergoing ENT surgery had OR cultures obtained and 45 (5.0%) of those had MRSA PCR collected within the same admission. The baseline demographic and clinical characteristics of these 45 patients are described in Table 1. Twenty-seven (60%) were men and 18 (40%) were women and mean age was 52 (± 18 SD) years. The most common source of infection was abscess. Median time between MRSA PCR collection and OR culture collection was 1 day (IQR -1, 4 days).

**Table 1.** Clinical characteristics of patients

	N = 45
Age, years (N, %)	
18–29	7 (15.6)
30–49	9 (20)
50–69	21 (46.7)
70+	8 (17.8)
Sex (N, %)	
Male	27 (60)
Female	18 (40)
Comorbidities (N, %)	
Hypertension	22 (48.9)
T2DM	16 (35.6)
Heart failure	4 (8.9)
COPD	3 (6.7)
CAD	2 (4.4)
Source (N, %)	
Abscess	24 (53.3)
Sinusitis	8 (17.8)
Trauma	4 (8.9)
SSI	3 (6.7)
Osteomyelitis	2 (4.4)
Other^a^	4 (8.9)
Time from MRSA nasal PCR collection to culture collection (days, +/-IQR)	1 (+/-1)

Note. T2DM, type 2 diabetes mellitus; COPD, chronic obstructive pulmonary disease; CAD, coronary arterial disease; SSI, surgical site infection.

a Necrotizing fasciitis, esophageal mass, trachea-esophageal fistula repair, and tracheal stenosis repair.

Forty (89%) patients had a positive OR culture; 9 (20%) monomicrobial, and 31 (69%) polymicrobial. *Staphylococcus aureus* was isolated from 6 (13%) patients and of these, only 2 (4%) were MRSA. The most common organism isolated was Anginosus group Streptococcus followed by *Staphylococcus epidermidis*. Five (11%) patients were colonized with MRSA based on PCR screening.

Of patients with a negative MRSA PCR, none of the OR cultures grew MRSA indicating a NPV of 100%. The diagnostic characteristics of MRSA PCR are shown in Table 2.

**Table 2.**
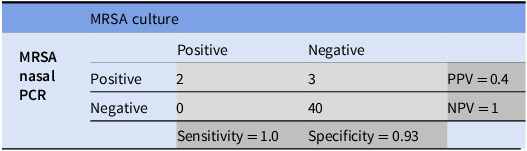
Diagnostic characteristics of MRSA nasal PCR

## Discussion

This study adds to a large body of work exemplifying the diagnostic utility of MRSA PCR testing. These results reveal that nasal MRSA PCR has a remarkably high NPV for MRSA infection in head and neck infections. The present study is novel due to the patient level detail provided. Previous studies have largely been based on population data and lack the specifics of culture data beyond the anatomic location of ENT.^6^ Data regarding patient age, sex, infectious source, and comorbidities were extracted along with data on the specific surgical intervention performed. These data provide a correlation between the performance of this diagnostic test and specific patient groups and surgical interventions. This degree of detail is currently absent in the literature and should stimulate further studies in a larger, prospective manner.

Limitations for the present study are inherent to the study design in that it was performed at a single center with a small sample size. Additionally, there were only two patients who had MRSA identified in culture in our study population, and it is unknown if the exceptional NPV would remain if more samples were collected. With the low incidence of MRSA in our study population and our small sample size, this study is likely underpowered; however, given the retrospective nature of this study, sample size calculations were not performed. *Staphylococcus aureus* was isolated less frequently than Anginosus group Streptococci, S*taphylococcus epidermidis*, and anerobic organisms which is consistent with previously established microbiologic data of head and neck infections.^8^ There was heterogeneity among patients and source of infection; however, the strict inclusion criteria presented a group of individuals with similar interventions in the same location of the body. In instances when providers have no culture data to base their decisions, antibiotic selection is often based on location and known bacterial colonization and as such the presented group of patients is clinically uniform. At our institution, there is not an established role for MRSA PCR in the perioperative setting. Utilization was based on clinician discretion which is a possible avenue to introduce selection bias. Vancomycin was started in 38 (84%) of patients included in the study, and 22 (49%) were initiated prior to going to the OR. As such, our MRSA culture results may be confounded by prior vancomycin administration. Depending on a patient’s clinical stability, it is often unreasonable to withhold antibiotics. It is this situation in which MRSA PCR testing can provide benefit to the clinician when deciding to start empiric antibiotics. In this small retrospective study, nasal MRSA PCR testing had a NPV of 100% for isolation of MRSA from ENT operative cultures and could be helpful in determining empiric antibiotic selection.
